# Drug‐grafted DNA as a novel chemogene for targeted combinatorial cancer therapy

**DOI:** 10.1002/EXP.20210172

**Published:** 2022-03-22

**Authors:** Yuhe Liu, Jiao Zhang, Yuanyuan Guo, Ping Wang, Yue Su, Xin Jin, Xinyuan Zhu, Chuan Zhang

**Affiliations:** ^1^ School of Chemistry and Chemical Engineering, Frontiers Science Center for Transformative Molecules, Institute of Molecular Medicine, Sixth people's Hospital, School of Medicine, Shanghai Key Laboratory for Molecular Engineering of Chiral Drugs Shanghai Jiao Tong University Shanghai China; ^2^ Department of Radiology Shanghai Jiao Tong University Affiliated Sixth People's Hospital Shanghai Jiao Tong University School of Medicine Shanghai China; ^3^ Weigao Research Center Shanghai China

**Keywords:** antisense oligonucleotide, cancer therapy, chemogene, DNA‐drug conjugate, drug delivery system, phosphorothioate DNA

## Abstract

Combinatorial therapy based on chemotherapeutic drugs and gene agents to achieve synergistic antitumor effects has emerged as a new direction for cancer treatment. However, simple and efficient co‐delivery of those two drug categories remains a key challenge in this hot area owing to their substantially different pharmacodynamics, impeding the translational potentials of combinatorial approaches. To address this issue, herein we propose a simple strategy to site‐specifically graft camptothecins (CPTs, a representative chemodrug) onto a DNA with dual functional segments, including an AS1411 aptamer sequence to target the cancer cell and a BCL‐2 antisense sequence to down‐regulate the anti‐apoptotic gene. The obtained DNA‐drug conjugate possesses precise chemical composition, controllable drug loading ratio, and responsive disulfide linkage, which can serve as a novel type of chemogene for combinatorial cancer therapy. In both in vitro and in vivo evaluations, our CPT‐bearing chemogene exhibit the targeted co‐delivery of chemo and gene agents to tumor site, efficient BCL‐2 gene knockdown, and strong induced apoptosis of cancer cells, together leading into an enhanced antitumor efficacy. With simple and precise structure as well as facile synthetic procedure, the new chemogene may turn into a promising drug formulation for combinatorial antitumor treatment.

## Graphical abstract

A new type of targeted chemogene is constructed through site‐specific grafting camptothecins onto a phosphor othioate DNA containing antisense and aptamer segments. With simple and precise structure, the targeted chemogene shows hallmark properties in both in vitro and in vivo evaluations, including active targeting capability, vigorous gene regulation pitency, and prominent antiumor effect, providing new potentials for a better cancer therapy.

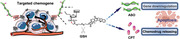

## 1 INTRODUCTION

Despite great advances achieved in cancer therapy, as one of the most deadly diseases in the world, there is still lack of effective curing strategies for wide types of malignant tumors in clinical practices.^[^ ^1^, ^2^ ^]^ Particularly, it is often difficult to eliminate the whole tumor and prevent the metastasis of the tumor cells with a single treatment mode due to the complexity, diversity, and heterogeneity of the tumor itself.^[^ ^3^ ^]^ In cancer treatment, monotherapy that relies on a single therapeutic agent and modality often exhibits low response rate and unsatisfied antitumor efficacy as the tumors may rapidly adapt the environmental change during the treatment, thus causing the resistance to used drug.^[^ ^4^, ^5^, ^6^, ^7^ ^]^ For instance, in the course of chemotherapy, the continuous application of chemotherapeutic drugs usually causes multidrug resistance of tumors rapidly.^[^ ^8^ ^]^ In radiotherapy, tumor cells in hypoxic areas are less sensitive to radiotherapy, preventing tumor tissue from being completely killed.^[^ ^9^ ^]^ As such, recently researchers have put large efforts to develop combinatorial drug delivery systems which can not only integrate the advantages of each therapeutic modality, but also strengthen the therapeutic effect in a synergistic way, thus reducing the drug dosage and side effects.^[^ ^10^ ^]^

In combinatorial therapies against cancer, nucleic acid therapeutics including antisense oligonucleotides (ASOs) and small interfering RNAs (siRNAs)^[^ ^11^, ^12^, ^13^ ^]^ are frequently employed to suppress the expression of tumor‐driven oncogenes or drug‐resistance‐related proteins, which can further enhance the sensitivity and potency of chemotherapeutics to achieve additive therapeutic effects.^[^ ^14^, ^15^, ^16^ ^]^ Despite great advances, co‐delivery of chemo and gene therapeutics for combinatorial therapy still encounters limitations, including unstable drug packages, potential separation of the drugs during the circulation, and the lack of precise control over the gene and chemo drug ratio.^[^ ^17^, ^18^, ^19^, ^20^ ^]^ Meanwhile, exogenous materials (e.g. synthetic polymers, inorganic nanoparticles) based co‐delivery systems commonly used in previous studies on one hand increase the complexity of the formulation, on the other hand also raise biosafety concerns for gene and chemodrug delivery.^[^ ^21^, ^22^ ^]^ Besides performing as genetic biomolecule in living systems,^[^ ^23^ ^]^ DNA has also emerged as carrier material to co‐deliver gene agents and other functional therapeutics, such as small molecular drug and protein, which exhibited excellent antitumor efficacy.^[^ ^24^, ^25^ ^]^ Recently, we developed a two‐in‐one “chemogene” construct, in which therapeutic nucleoside analogs (NAs) were directly integrated into an antisense strand through solid‐phase synthesis.^[^ ^26^ ^]^ Different from conventional co‐delivery complexes that contain multiple agents with varied roles, chemogene is a single entity with dual functions which eliminates the pharmacodynamic difference of chemo and gene agents, providing unique advantages in combinatorial antitumor therapy. However, the type of drug that can be integrated in chemogene is limited since most clinically used chemodrug can be easily converted into phosphoramidate for solid‐phase synthesis. To solve this issue, there is an unmet need to develop a more general method to construct novel chemogenes beyond the NA. Meanwhile, similar to DNA strands, NA‐integrated chemogene is a highly negatively charged biomacromolecule that has difficulty entering the cells. In our previous exploration, we conjugate the NA‐integrated chemogene with a polycaprolactone to form a spherical nucleic acid for efficient cellular internalization.^[^ ^26^ ^]^ However, micellular SNA constructs often lack control in composition, size distribution, and targeting ability. Therefore, simple and precise structure as well as the active targeting capability should be taken into account in the design of non‐NA chemogene.

**SCHEME 1:**
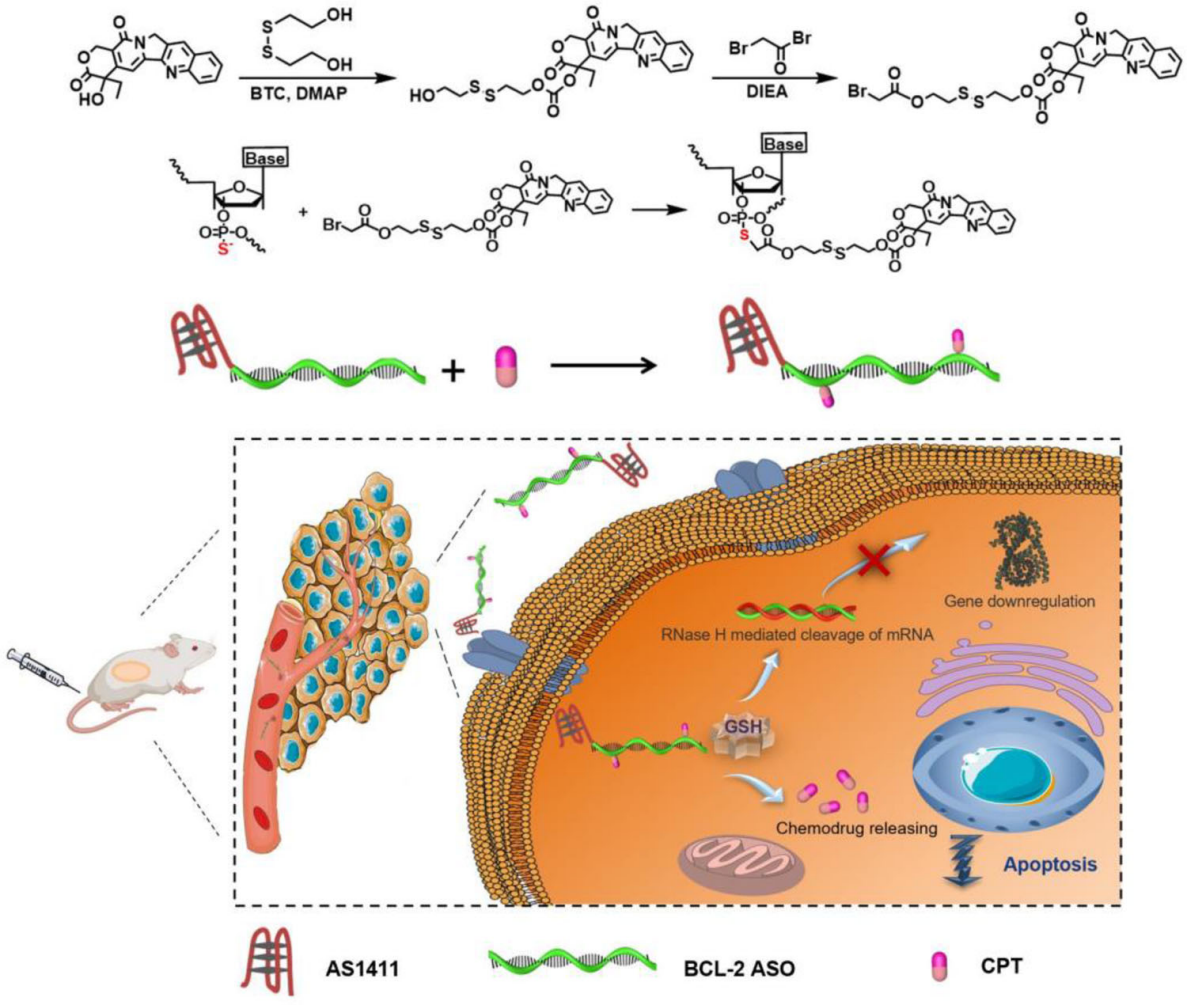
Schematic illustration of the synthetic route of the CPT‐bearing chemogene with targeting capability and its co‐delivery process as precise and carrier‐free nanomedicine

To achieve the goals mentioned above, herein we report a strategy that can precisely graft the commonly used chemodrug on phosphorothioate (PS) modified ASO to generate a new type of chemogene (Scheme 1). As a proof‐of‐concept study, antisense sequence (G3139) that targets BCL‐2 gene, a key regulatory gene to inhibit apoptosis of cancer cells,^[^ ^27^, ^28^ ^]^ was selected and two PS modifications were introduced to the ASO sequence for drug grafting. The nucleophilic feature of PS group allows the convenient conjugation of drug molecules pre‐modified with electrophilic moieties, such as carbonethyl bromide group or benzyl bromide group.^[^ ^29^, ^30^ ^]^ In this study, we chose camptothecin (CPT)^[^ ^31^ ^]^ as a representative non‐NA chemodrug that was first modified with a carbonethyl bromide group following our previous study.^[^ ^32^, ^33^ ^]^ Thereafter, two CPTs can conveniently graft onto DNA sequence at PS‐modified sites, generating a new type of chemogene. Moreover, to enable the new chemogene with targeting capability, an aptamer sequence (AS1411) is introduced and directly extended at the 5′ terminus of antisense strand, allowing us to construct a targeted CPT‐bearing chemogene. Note that, a disulfide bond is also introduced between CPT and DNA, which further endows the chemogene redox responsive feature and facilitates the drug release in tumor cells. With the obtained new chemogene, its precise drug‐loading characteristics, cellular uptake behavior, targeting delivery feature, gene regulatory capability, and antitumor efficacy were systematically evaluated in both in vitro and in vivo experiments, illustrating its great potentials for combinatorial cancer treatment.

## 2 RESULTS AND DISCUSSION

### 2.1 Syntheses and characterizations of CPT‐grafted ASOs as new chemogenes

**FIGURE 1:**
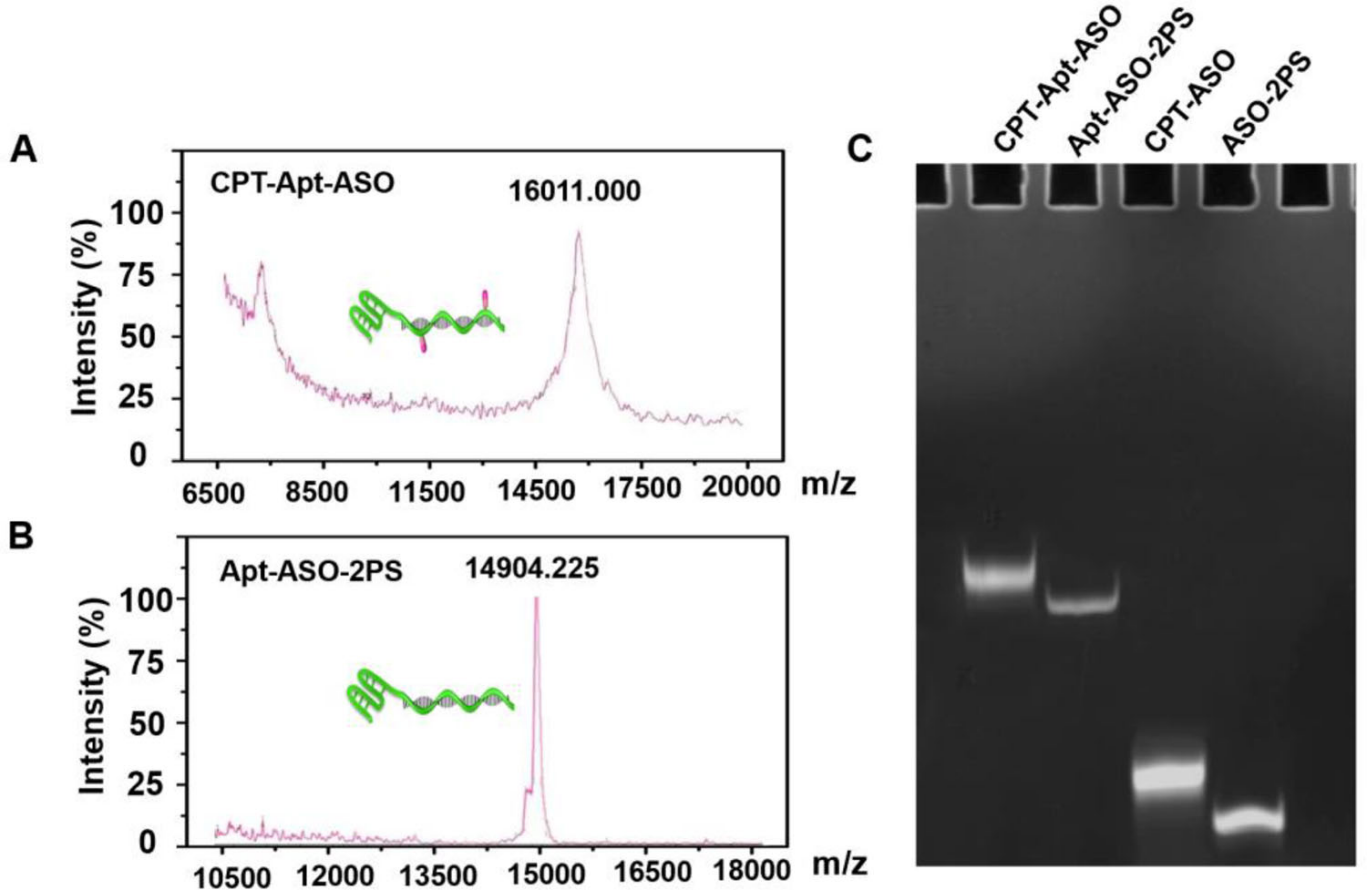
Synthesis and characterizations of CPT‐grafted oligonucleotides. (A,B) MALDI‐TOF spectra of aptamer‐containing antisense oligonucleotides before (Apt‐ASO‐2PS) and after CPT (CPT‐Apt‐ASO) grafting; (C) CPT‐grafted antisense oligonucleotides characterized by 20% denaturing polyacrylamide gel electrophoresis

To conjugate the CPT with predesigned DNA strands, we first modified the CPT with a carbonethyl bromide group following the protocol of our previous study (Scheme S1, see detail procedure in Supporting Information).^[^ ^32^ ^]^ Briefly, a bioreducible disulfide linker was first conjugated with CPT using triphosgene as activation agent to synthesize a disulfide‐modified CPT (CPT‐ss‐OH). Thereafter, the product was further mixed with bromoacetyl bromide to obtain the carbonethyl bromide‐modified CPT (CPT‐ss‐Br). For each step, the product was analyzed by nuclear magnetic resonance (NMR) spectroscopy, which confirmed the successful syntheses of CPT‐ss‐OH and CPT‐ss‐Br, respectively (Supporting Information, Figure S1A,B). Meanwhile, high purity and molecular weight of liquid chromatography (LC) and mass spectrometry (MS) also confirmed our synthesis of CPT‐ss‐Br (Figure S2). In this study, we specifically introduce one PS modification close to each termini of the ASO segment, which mimics the gapmer construct with a central segment maintaining the capability of RNase H mediated target mRNA degradation. As a systematical study, PS‐modified BCL‐2 ASO strands with or without aptamer segment (Apt‐ASO‐2PS and ASO‐2PS), scramble strand (Apt‐SC‐2PS) without gene regulatory capability were designed (see detail sequences in Table S1) to incubate with CPT‐ss‐Br, resulting in the CPT grafting on corresponding DNA strands and generations of targeted chemogene (CPT‐Apt‐ASO), non‐targeted chemogene (CPT‐ASO), and scramble CPT‐DNA conjugate (CPT‐Apt‐SC), respectively. After the syntheses, the obtained chemogenes were characterized by multiple techniques. As shown in Figure 1, matrix‐assisted laser desorption/ionization time‐of‐flight mass spectrometry (MALDI‐TOFMS) demonstrated the molecular weights of both CPT‐Apt‐ASO and Apt‐ASO‐2PS, difference between which was consistent with the mass of two CPT loadings on DNA strand, indicating that the complete drug‐grafted on two PS sites of the oligonucleotide. Moreover, the products were also analyzed by denaturing polyacrylamide gel electrophoresis (PAGE, 20% w/w). As shown in Figure 1C, sharp bands appeared after the conjugation with CPTs, which had significantly lower migration rate than those of initial PS‐modified ASOs. Note that, the obtained conjugates could completely run into the gel and no aggregates were observed, confirming the good solubility of the chemogene in aqueous condition. Moreover, the stability of chemogene was evaluated by incubating the samples with cell culture medium containing 10% fetal bovine serum (FBS) for varied times. As shown in Figure S3, the chemogene almost keep intact in gel electrophoresis and no obvious degradation, indicating the relatively good stability of chemogene under physiological condition (Figure S3).

### 2.2 Molecular recognition of CPT‐bearing chemogenes

ASO‐based gene knockdown highly relies on molecular recognition between ASO and its target mRNA to form a hybrid DNA/RNA duplex, which recruits the RNase H to degrade the RNA part.^[^ ^34^ ^]^ In our new chemogene, CPT drug is grafted on the backbone of DNA, which would not affect the basepairings between the modified DNA sequences and their target substrate, thus maintaining their antisense functions. To confirm the retained molecular recognition of CPT‐bearing chemogenes, the obtained CPT‐ASO and CPT‐Apt‐ASO were employed to rapidly anneal with their complementary strands in 1 × TAE/Mg^2+^ buffer, respectively. The non‐denaturing gel electrophoresis revealed that the duplex structures (CPT‐ASO‐bp and CPT‐Apt‐ASO‐bp) could successfully be formed as sharp bands with much slower mobility appeared after annealing, indicating the retained hybridization capability of the chemogene to their complementary target sequences (Figure S4).

### 2.3 In vitro cellular uptake behaviors of CPT‐bearing chemogenes

**FIGURE 2:**
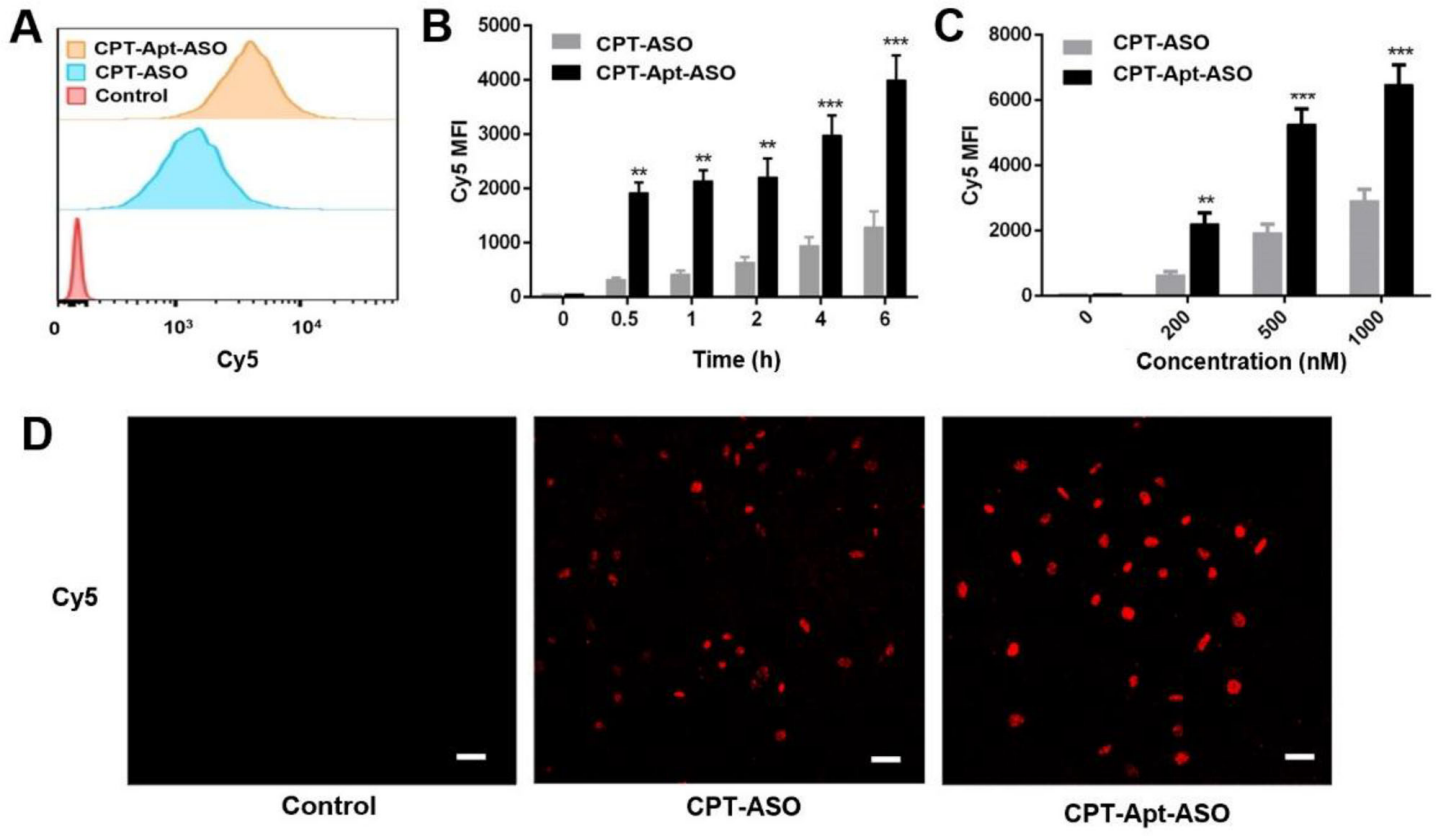
The intercellular behaviors of CPT‐bearing chemogenes. (A) In vitro cellular uptake behaviors of CPT‐ASO and CPT‐Apt‐ASO (equivalent Cy5 concentration: 0.5 μM) determined by flow cytometry analysis. (B,C) The mean fluorescence intensities of cells treated with CPT‐ASO and CPT‐Apt‐ASO for different incubation times and concentrations. (D) CLSM images of HeLa cells treated with Cy5‐labeled CPT‐ASO and Cy5‐labeled CPT‐Apt‐ASO for 2 h. Blue: Hoechst (nuclear stain); red: Cy5. Scale bars: 50 μm

After characterizations, cellular uptake behaviors of the new chemogenes were evaluated by incubating them with HeLa cells and then analyzed by flow cytometry and confocal laser scanning microscopy (CLSM). It is well established that AS1411 aptamer can sequence specifically target the overexpressed nucleolin protein on tumor cell surface.^[^ ^35^ ^]^ To demonstrate the targeting capability upon equipping the chemogene with aptamer, both CPT‐ASO and CPT‐Apt‐ASO were labeled with Cy5 to provide fluorescence signals. As shown in Figure 2A, compared to mock group, both CPT‐ASO and CPT‐Apt‐ASO treated groups exhibited strong fluorescence after incubation. Furthermore, mean fluorescence intensities of the cells significantly increase along with the incubation time and chemogene concentration (Figure 2B,C). Specifically, fluorescence intensities of the cells treated with CPT‐Apt‐ASO were always significantly higher than that of CPT‐ASO treated one at all incubation time and concentration, indicating that the aptamer introduction truly endows the chemogene targeting capability and increases the cellular uptake efficiency. A similar result can be found in CLSM imaging, in which targeted chemogene treated cells show the strongest fluorescent signals (Figure 2D). Based on results above, we can confirm that our aptamer‐equipped chemogene has specific tumor‐targeting capability, which may perform as a good candidate for targeted drug delivery.

### 2.4 Cell apoptosis and gene knockdown induced by CPT‐bearing chemogene

**FIGURE 3:**
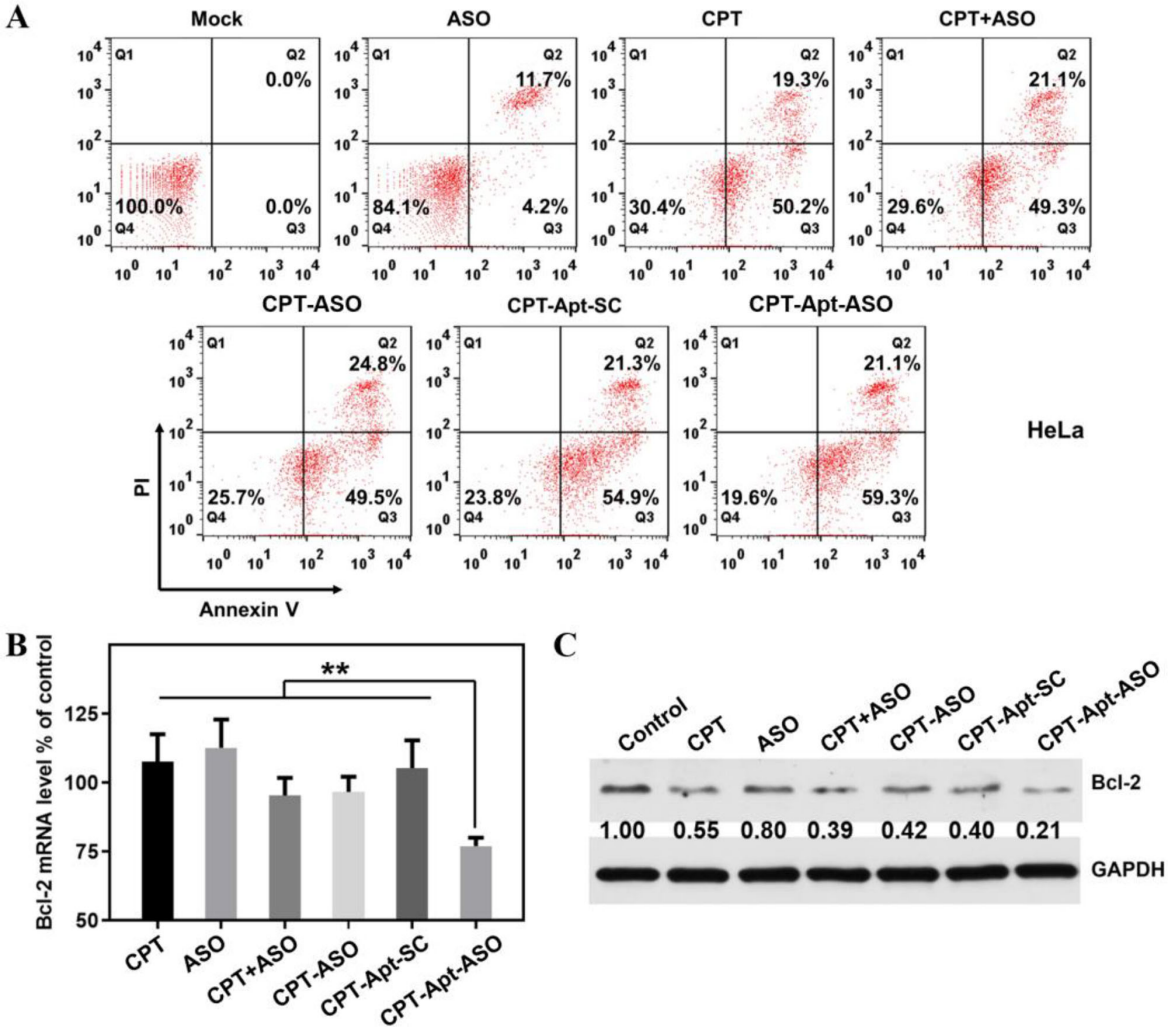
Cell apoptosis and gene knockdown induced by CPT‐bearing chemogenes. (A) Flow cytometry‐based apoptosis analysis of HeLa cells incubated with CPT, ASO, CPT+ASO, CPT‐ASO, CPT‐Apt‐SC, and CPT‐Apt‐ASO at an equivalent CPT concentration of 0.2 μM for 48 h incubation. (B,C) BCL‐2 gene expression after incubating with CPT, ASO, CPT+ASO, CPT‐ASO, CPT‐Apt‐SC, and CPT‐Apt‐ASO, respectively. mRNA and protein levels of BCL‐2 expression were determined by qRT‐PCR analysis and western blot assay respectively. Statistical significance: *p < 0.05, **p < 0.01, and ***p < 0.001

With enhanced cellular uptake efficiency of CPT‐bearing chemogenes, their antitumor effects were further evaluated by flow cytometry‐based apoptosis assay for both non‐targeted and targeted chemogenes. After 48 h incubation of the HeLa cells with different drug formulations, apoptosis rates induced by CPT, ASO, CPT+ASO, CPT‐ASO, CPT‐Apt‐SC, and CPT‐Apt‐ASO are 69.6%, 15.9%, 70.4%, 74.3%, 76.2%, and 80.4%, respectively (Figure 3A). These results demonstrate that CPT‐bearing chemogenes can strongly induce the apoptosis of cancer cells, thus implying their good antitumor capability for cancer treatment. Particularly, we can notice that the targeted chemogene group showed the highest induced apoptotic rate compared to those of non‐targeted chemogene and other groups, which may be ascribed to its aptamer‐mediated targeted delivery feature and enhanced cellular internalization. Moreover, we also applied the CPT‐bearing chemogenes to incubate with drug‐resistant cancer cells. Similarly, for both drug‐resistant A549 and BEL‐7402 cells, the targeted CPT‐Apt‐ASO showed the strongest tumor‐killing capability as confirmed by the highest induced apoptotic rates of corresponding cancer cells (Figure S6). According to the results above, we can conclude that our new chemogenes especially the one with targeting capability can exert strong antitumor effect on both regular and drug‐resistant cancer cells.

The promoted cytotoxicity of chemogenes cannot barely be ascribed to its enhanced cellular uptake efficiency but also owing to its capability of gene regulation. To verify this, the target gene knockdown induced by CPT‐bearing chemogene was evaluated at both mRNA and protein levels. As shown in Figure 3B, CPT and ASO alone exhibit almost no knockdown effects at mRNA level since ASO itself cannot internalize into the cells easily. In contrast, the targeted chemogene group (CPT‐Apt‐ASO) shows an obvious down‐regulation of BCL‐2 with the highest efficiency. Meanwhile, the non‐targeted CPT‐ASO and CPT+ASO mixture demonstrates only trivial knockdown of BCL‐2 expression, which may be attributed to the slightly promoted ASO uptake after being grafted or mixed with CPT. Note that, when changing the ASO to a scramble sequence, there is no obvious knockdown effect regarding BCL‐2 expression, indicating the down‐regulation induced by chemogene is a sequence‐specific process. Besides the confirmation of gene knockdown at mRNA level, reduced BCL‐2 protein expression was also verified by western blot analysis. As shown in Figure 3C, a similar trend can be observed for BCL‐2 protein expression after incubation with CPT‐bearing chemogene and control samples where CPT‐Apt‐ASO group shows the highest knockdown of BCL‐2 protein, which is consistent with qRT‐PCR results. The efficient down‐regulation of anti‐apoptotic BCL‐2 protein would facilitate the tumor cell apoptosis and restore the sensitivity of cancer cells to chemodrug, which lays the foundation for its combinatorial cancer treatment applied in vivo.

### 2.5 Biodistribution of CPT‐bearing chemogene

**FIGURE 4:**
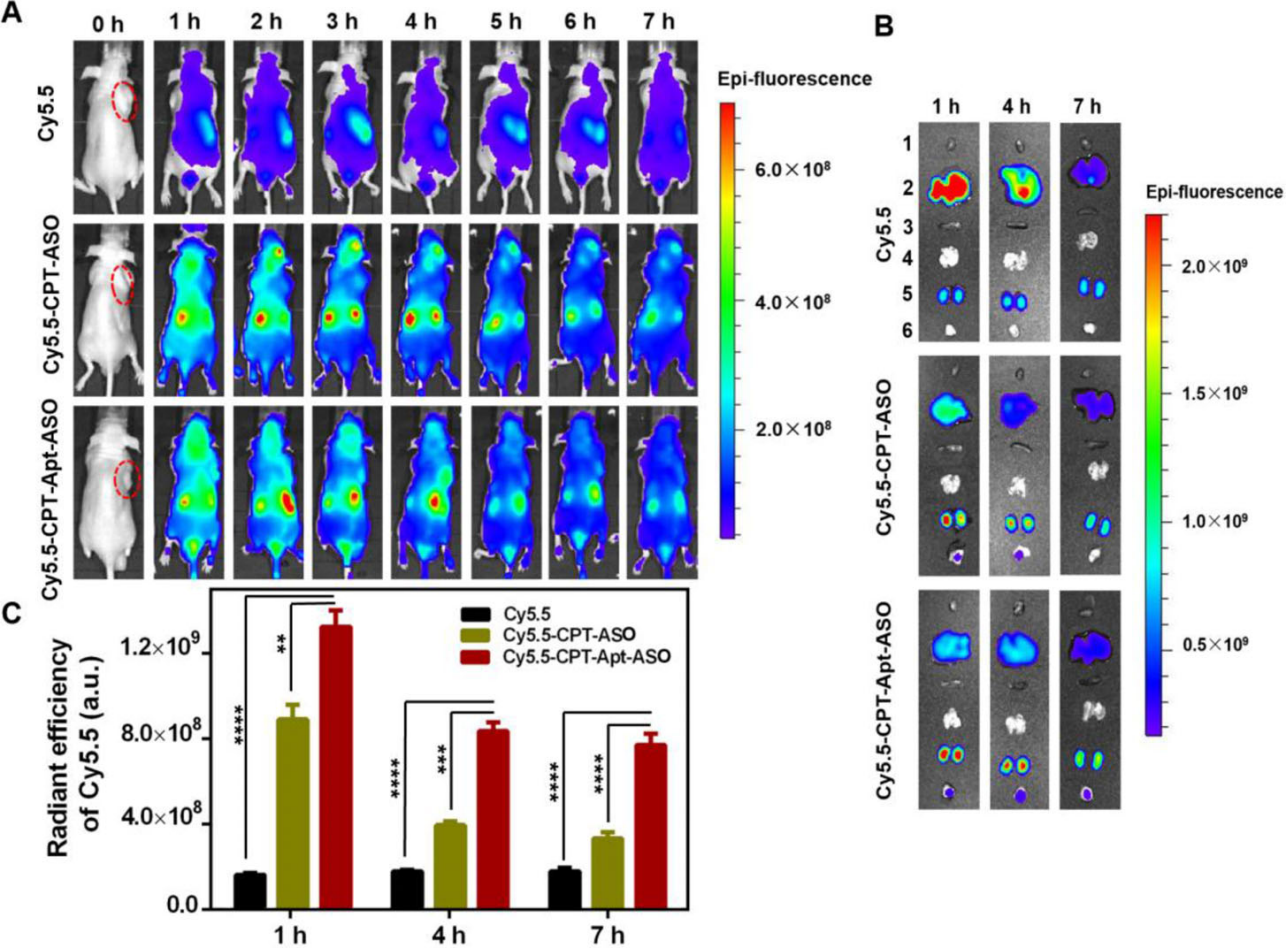
The biodistributions of CPT‐bearing chemogenes. (A) In vivo images of HeLa tumor‐bearing nude mice treated with Cy5.5, Cy5.5‐labeled CPT‐ASO, and Cy5.5‐labeled CPT‐Apt‐ASO at 1, 2, 3, 4, 5, 6, and 7 h post injection. Red‐dashed circles indicate the tumor sites. (B) Fluorescent images of major organs and tumors obtained from the HeLa tumor‐bearing nude mice treated with Cy5.5, Cy5.5‐labeled CPT‐ASO, and Cy5.5‐labeled CPT‐Apt‐ASO at 1, 4, and 7 h. 1: heart, 2: liver, 3: spleen, 4: lung, 5: kidneys, 6: tumor. (C) Quantitative analysis of the tumor accumulation of Cy5.5‐CPT‐ASO and Cy5.5‐CPT‐Apt‐ASO at 1, 4, and 7 h post injection. Statistical significance: *p < 0.05, **p < 0.01, ***p < 0.001, and ****p < 0.0001. Asterisks represent statistical significance between the mice treated with CPT‐Apt‐ASO and other drug formulations

Based on the good performance of new chemogene in vitro, we further investigate its in vivo pharmacodynamic properties. First, in vivo targeting capability of the chemogene was demonstrated by biodistribution study on a tumor‐bearing mouse model. Thus, both targeted and non‐targeted CPT‐bearing chemogenes were labeled with Cy5.5. Meanwhile, free Cy5.5 was also used as control. All samples were intravenously administrated into HeLa tumor‐bearing mice and the sample distribution of each group was recorded using an IVIS Lumina II in vivo imaging system at 1, 2, 3, 4, 5, 6, and 7 h post injection. As shown in Figure 4A, with aptamer as an active targeting moiety, Cy5.5‐CPT‐Apt‐ASO shows strong fluorescent signals in tumor tissue at 1 h post injection, which is further increased from 2 to 4 h, and then weakened from 5 to 7 h post injection, demonstrating an obvious accumulation of targeted chemogene at the tumor site. In contrast, only weak fluorescent signals can be observed for the free Cy5.5 treated group at the tumor site, implying that the small molecular drug is rapidly cleared from the circulation after injection. Additionally, non‐targeted Cy5.5‐CPT‐ASO shows tumor accumulation in some extent but the fluorescent signal is weaker than that of targeted chemogene at 2–4 h post injection. Thereafter, no obvious fluorescent signal can be observed at the tumor site for non‐targeted group (Figure 4A).

To further validate the accumulation of CPT‐bearing chemogene in tumor and its distribution in major organs, tumor‐bearing mice were intravenously injected above three formulations again. At 1, 4, and 7 h post injection, the tumor tissues, and major organs were dissected from those three groups of mice and imaged by in vivo fluorescence imaging system. As shown in Figure 4B, free Cy5.5 fluorescent molecule is mainly concentrated in the liver and kidney at those three pre‐determined time points, and the fluorescent signals in the tumor site are extremely low. Comparatively, Cy5.5‐CPT‐Apt‐ASO treated group shows obvious enriched fluorescent signals in tumor tissues even at 7 h post injection, which is consistent with the results of in vivo imaging mentioned above. Meanwhile, lower fluorescent signals can be observed at the tumor site for Cy5.5‐CPT‐ASO treated group. However, the fluorescence vanishes more rapidly than the targeted chemogene treated group and there is almost no fluorescent signal in the tumor tissue at 7 h post injection (Figure 4B). With ex vivo fluorescence images of dissected tumors, the quantitative analysis of the chemogene accumulation at the tumor site is shown in Figure 4C. Based on the quantitative results, we can see that the strongest fluorescence intensity appears at 1 h post injection for both targeted and non‐targeted chemogenes and fluorescence intensities of Cy5.5‐CPT‐Apt‐ASO group are about twice than that of Cy5.5‐CPT‐ASO group at all time points (Figure 4C). All these results demonstrate that CPT‐bearing chemogene with aptamer segment has active targeting capability in vivo.

### 2.6 In vivo antitumor effect of CPT‐bearing chemogenes

**FIGURE 5:**
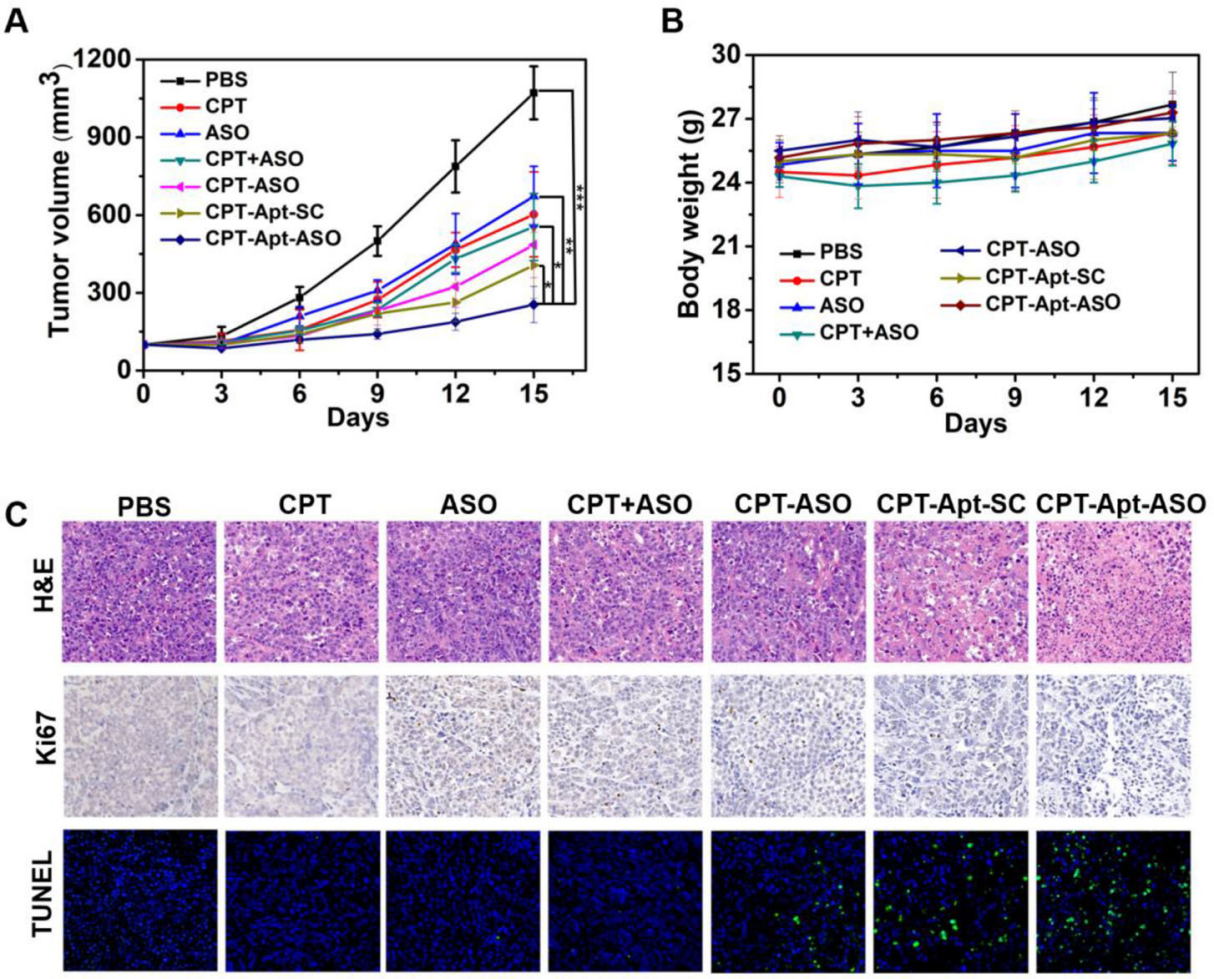
In vivo antitumor effects of CPT‐bearing chemogenes. (A) The tumor growth curves of mice treated with CPT, ASO, CPT+ASO, CPT‐ASO, CPT‐Apt‐SC, and CPT‐Apt‐ASO at an equivalent dose of CPT (3.5 mg/kg) every 3 days for five times (*n* = 3 in each group). Statistical significance; *p < 0.05, **p < 0.01, and ***p < 0.001. Asterisks represent statistical significance between the mice treated with CPT‐Apt‐ASO and other drug formulations. (B) Bodyweight changes of mice in different treatment groups. Data were shown as mean ± SD. (C) Histological and immunohistochemical staining of tumor sections after the treatment with different formulations

With accumulated evidence of effective targeting capability and enhanced cytotoxicity in vitro, in vivo antitumor efficacy of the new chemogenes was evaluated on subcutaneous tumor‐bearing mouse model. As such, HeLa subcutaneous tumor models were established and the tumor‐bearing mice were injected with PBS, CPT, ASO, CPT+ASO, CPT‐ASO, CPT‐Apt‐ASO, and CPT‐Apt‐SC once every 3 days respectively. As shown in Figure 5A, CPT‐Apt‐ASO demonstrates as the most efficient one to inhibit tumor growth compared with the control groups of PBS, CPT, ASO, CPT+ASO, CPT‐ASO, and CPT‐Apt‐SC, whereas tumor size in PBS group increases rapidly. Similarly, the survival status of mice during the treatment also proves that our chemogenes have no obvious toxicity and can be well tolerated by treated mice ((Figure 5B). Sacrificing the mice after the fifth treatment, the dissected tumors were imaged and shown in Figure S8. Compared with the control group, the tumor size of the CPT‐Apt‐ASO treated group is the smallest on average, indicating that the targeted CPT‐bearing chemogene has the best antitumor effect in the mouse model.

Finally, strong antitumor effects induced by CPT‐bearing chemogene were further verified through histological and immunohistochemical analysis. Hematoxylin and eosin (H&E) staining reveals that cells obtained from tumors tissue in the PBS groups have well‐defined cytoplasm and nuclei and at the meantime CPT, ASO, CPT+ASO, CPT‐ASO, and CPT‐Apt‐SC groups show some sporadic nuclear shrinkages (pyknosis), indicating slightly induced cell damages (Figure 5C, upper panel). In contrast, more obvious nuclear shrinkage, tissue destruction, and a certain degree of nuclear loss could be found in the CPT‐Apt‐ASO‐treated group. Moreover, Ki67 immunohistochemical staining reveals the lowest brown Ki67 signals in the CPT‐Apt‐ASO group, indicating that the proliferation of tumor cells is significantly inhibited by the targeted chemogene (Figure 5C, middle panel). Meanwhile, the terminal transferase‐mediated dUTP nick end‐labeling (TUNEL) staining also demonstrates that the green fluorescent signals in nuclei of the CPT‐Apt‐ASO treated group are significantly stronger than those of PBS, CPT, ASO, CPT+ASO, CPT‐ASO, and CPT‐Apt‐SC groups, illustrating higher apoptosis of tumor cells induced by our targeted chemogene. Lastly, histological examinations of the major organs of mice after treatment are shown in Figure S9. For all treated groups, no obvious tissue damages can be observed for heart, liver, spleen, lung, and kidneys, again confirming the negligible systemic toxicity of administrated drugs. Taken together, our new chemogene shows a significantly higher antitumor efficacy in vivo compared to individual chemo or gene drug used alone, providing new potentials to realize a better combinatorial cancer therapy.

## 3 CONCLUSION

We construct a new type of chemogene with active targeting capability by simply grafting small chemodrugs on the backbone of functional DNA strand. The obtained chemogene is a single molecular entity but contains an aptamer as targeting moiety, two CPT molecules as chemotherapeutics, and an antisense sequence as gene regulatory agent. With precise structure and multitude of functions, our new chemogene exhibits the targeted drug delivery, efficient gene regulation capability, and enhanced synergistic antitumor efficacy both in vitro and in vivo. Compared to NA‐integrated chemogene developed in our previous work,^[^ ^27^ ^]^ the reported drug‐grafted DNA here represents the first non‐NA constituted chemogene for combinatorial cancer therapy. As a general approach, CPT can be easily changed to other commonly used chemotherapeutics, which can extremely expand the scope of drug for chemogene engineering. Meanwhile, the sequences of antisense and aptamer segments in chemogene can also be conveniently designed to target other disease‐related genes or substrates on cell surface. Along with its simplicity on chemical structure and synthetic route, chemogene with dual and synergistic functions may have great potentials on the way forward for better cancer therapy.

## 4 MATERIALS AND METHODS

### 4.1 Materials

PS oligonucleotides were purchased from Sangon Biotech Co., Ltd. (Shanghai, China). CPT was purchased from Dalian Melune Biotechnology Co., Ltd. (China). Annexin V‐FITC apoptosis assay kit for flow cytometry was purchased from Thermo Fisher Scientific (Waltham, MA, USA).

### 4.2 Syntheses and characterizations of CPT‐grafted oligonucleotides

To conjugate the CPT on ASO strands, intermediate compound disulfide‐modified CPT (CPT‐ss‐OH) and carbonethyl bromide‐modified CPT (CPT‐ss‐Br) were synthesized according to our previous work (Supporting Information, Scheme S1).^[^ ^32^, ^33^ ^]^ Then, the obtained CPT‐ss‐Br was mixed with the ASO‐2PS (250 mΜ) at a predetermined ratio (PS group/bromide group = 1:20) in dimethyl sulfoxide solution. The reaction was kept on a roller at 50 °C for 1 h. The excess CPT‐ss‐Br was removed multi‐round extracted by ethylene acetate. CPT‐grafted PS ASO was characterized by 20% denaturing gel that contains 20% polyacrylamide (19:1 acrylamide/ bisacrylamide) and 8 M urea in a Tris‐Borate EDTA (TBE) buffer (89 mM Tris, 89 mM boric acid, and 2 mM EDTA). To evaluate the stability of new chemogene, we incubated the samples in cell culture medium containing 10% FBS at 37 °C for variable time intervals. After 2, 4, 6, 8, 10, and 12 h incubation, a 10 μl aliquot sample solution was transferred to plastic tubes and mixed with 2.5 mM EDTA solution to disable the enzyme activity and sample dispersed in PBS without treatment was used as a control. Then all samples were analyzed by 20% denaturing polyacrylamide gel electrophoresis and 1% agarose gel. The gel was stained with ethidium bromide (Thermo Fisher Scientific Inc., Carlsbad, CA, USA) and imaged by a fluorescence imaging system (Bio‐Rad Laboratories Co., Ltd., Hercules, CA, USA). Similarly, we also incubated the samples with different concentrations of DNase Ⅰ (5, 10, and 50 U/ml) and 20 U/ml DNase Ⅱ at 37 °C for fixed‐time essay and analyzed by 10% polyacrylamide gel electrophoresis.

### 4.3 Molecular recognition capability of CPT‐ASO and CPT‐Apt‐ASO

CPT‐ASO and CPT‐Apt‐ASO were equimolar (20 μM) mixed with their complementary strands in a TAE/Mg^2+^ (40 mM Tris, 2 mM EDTA•Na•H_2_O, 20 mM acetic acid, 12.5 mM (CH_3_COO)_2_Mg•_4_H_2_O, pH = 7.4, adjusted by acetic acid) buffer. The solution was incubated at 90 °C for 10 min and then cooled to 4 °C. The hybridization of CPT‐ASO and CPT‐Apt‐ASO with their complementary strands was examined by non‐denaturing polyacrylamide gel electrophoresis in a TAE/Mg^2+^ running buffer with 4% acrylamide (19:1 acrylamide/bisacrylamide) on a FB‐VE10‐1 electrophoresis unit.

### 4.4 Cell culture

Human colon cancer cells HCT116, drug‐resistant human liver carcinoma cells BEL‐7402, and drug‐resistant human non‐small cell lung cancer A549 were cultured at 37 °C in a humidified atmosphere containing 5% CO_2_. HCT116, drug‐resistant BEL‐7402, and drug‐resistant A549 cells were cultured in McCoy's 5A medium, Dulbecco's Modified Eagle's medium (DMEM), and RPMI‐1640 medium containing 10% FBS and antibiotics (50 units/ml penicillin and 50 units/ml streptomycin), respectively.

### 4.5 In vitro cellular uptake

We explored the specific targeting of CPT‐Apt‐ASO, and CPT‐ASO was employed as a control. The cellular uptake of CPT‐ASO and CPT‐Apt‐ASO were evaluated by flow cytometry and CLSM. For the flow cytometry study, HeLa cells were seeded at a density of 5 × 10^4^ cells per well in 24‐well plates in 1 ml culture medium and incubated at 37 °C overnight. Cy5‐labeled CPT‐ASO and CPT‐Apt‐ASO with equivalent Cy5 concentrations (0.2, 0.5, and 1 μM) were incubated with MCF‐7 and HeLa cells for predetermined time intervals (0.5, 1, 2, 4, and 6 h). Afterward, removing the culture medium, and cells were washed with PBS three times. Then, cells were detached using trypsin and collected cells were washed three times with PBS. Finally, the mean fluorescence intensities of cells were determined by BD FACSDiva software, and data for 1 × 10^4^ gated events were collected utilizing an FCM on an LSRFortessa (BD FACSCalibur, USA). For the CLSM study, HeLa cells were seeded at 4 × 10^4^ cells per well in 24‐well culture plates with a clean coverslip put in each well and grown overnight for attachment, followed by incubated with CPT‐Apt‐ASO labeled with Cy5 and CPT‐ASO labeled with Cy5 (equivalent Cy5 concentration: 0.5 μM) for 2 h. Subsequently, culture medium was removed, and cells were washed three times with PBS and fixed with 4% formaldehyde for 15 min at room temperature. The cells were stained with 1 μg/ml Hoechst 33342 solution for 15 min and the slides were rinsed with PBS three times, mounted, and observed with a laser scanning confocal microscope (Leica TCS SP8 STED 3X, Germany).

### 4.6 Cell apoptosis analysis

HeLa, A549/MDR, and BEL‐7402 cells were seeded in 12‐well plates at 3.0 × 10^5^ cells per well in 1 ml of Opti‐medium and cultured overnight. The cells were treated with CPT, ASO, CPT+ASO, CPT‐ASO, CPT‐Apt‐SC (a control sample using scramble sequence to replace the ASO segment in CPT‐Apt‐ASO), CPT‐Apt‐ASO, (0.2 μM CPT) for 48 h. For quantitative measurement of apoptosis, the treated cells were harvested and washed twice with ice‐cold PBS, stained with Alexa Fluor® 488 annexin V and PI, and no treatment group was used as control. Cell apoptosis was analyzed by flow cytometry (BD FACSCalibur, USA), and 1 × 10^4^ events per sample were counted according to the instructions.

### 4.7 Down‐regulation of BCL‐2 gene expression

The gene knockdown efficiency was evaluated by the corresponding mRNA and protein expression of BCL‐2. For mRNA detection, quantitative reverse transcription‐polymerase chain reaction (qRT‐PCR) assay was performed. First of all, the cells were cultured with CPT, ASO, CPT+ASO, CPT‐ASO, CPT‐Apt‐SC, CPT‐Apt‐ASO for 12 h. Then, fresh growth media were replaced for another 48 h incubation. Total RNA was extracted from cells by TRIzol extraction (Thermo Fisher Scientific Inc.). Untreated cells were used as negative control. Subsequently, the corresponding cDNAs were reverse‐transcribed using the AMV reverse transcription system (Promega) following the manufacture's procedure. The qRT‐PCR experiments were performed on a qRTPCR system (Tprofessional thermocycler, Biometra, Germany) using PowerUp™ SYBR® Green Master Mix kit (Thermo Fisher Scientific Inc., USA). The thermal cycling conditions for PCR program were: 95 °C for 3 min, 35 cycles of (95 °C for 60 s, 55 °C for 50 s, 72 °C for 60 s). Western blot assay was conducted to determine the BCL‐2 protein expression. After HeLa cells were treated with the drug formulations as described above, cells were lysed to extract the total proteins, which were assayed by immunoblotting to determine the expression levels of the BCL‐2 proteins. The total proteins were extracted from HeLa cells using RIPA buffer containing protease inhibitor cocktail. Thereafter, the protein concentrations were determined with S13 bicinchoninic acid (BCA) protein assay kit (Invitrogen, Carlsbad, CA, USA). Thereafter, 30 μg of proteins were separated by 5–15% SDS‐PAGE and transferred to 0.22 μm polyvinyl difluoride (PVDF) membranes (Millipore, USA). Subsequently, the membranes were blocked with 5% blotting grade milk, and then incubated with primary and secondary antibodies successively. After incubating with antibodies, the protein bands were imaged by electrochemical luminescence (Invitrogen, USA) and quantified by ImageJ software.

### 4.8 Biodistribution and in vivo optical imaging

All animal experiments were conducted per the guidelines of the Institution Animal Care and Use Committee of Shanghai Jiao Tong University. The study protocols involving animals were approved by the Animal Ethics Committee of Shanghai Jiao Tong University. HeLa tumor‐bearing nude mice (6 weeks old, male, Chinese Academy of Sciences of Shanghai) were prepared by subcutaneous injection of a suspension of 5 × 10^6^ HeLa cells in PBS (200 μl). In vivo optical imaging was performed when the tumor reached 300 mm^3^. Animals were intravenously injected free Cy5.5, Cy5.5‐labeled CPT‐ASO, and Cy5.5‐labeled CPT‐Apt‐ASO (with an equivalent Cy5.5 dose of 0.5 μmol/kg). At a predetermined time (1, 2, 3, 4, 5, 6, 7 h), mice were anesthetized using isoflurane/O_2_ (2% v/v) and images were taken and analyzed using an IVIS Lumina II in vivo imaging system (Caliper Life Sciences, USA) with 650 nm excitation wavelength and 700 nm emission wavelength. For the ex vivo biodistribution study, mice were sacrificed at 1, 4, and 7 h post injection. Major organs and tumor tissues were collected, rinsed by PBS, and imaged by IVIS Lumina II in vivo imaging system.

### 4.9 In vivo antitumor effects

Again, HeLa tumor‐bearing nude mice (6 weeks old, male) were prepared by subcutaneous injection of a suspension of 5 × 10^6^ HeLa cells in PBS (200 μl). When the size of tumor was reached at 100 mm^3^, mice began to be treated by intravenous injection of 200 μl of different drug formulations or PBS every 3 days for a maximum of five times (with an equivalent CPT dose of 3 mg/kg). Tumor volume and mouse weight were monitored every 3 days. The volume of each tumor was calculated using the following formula: volume = (length × width^2^)/2.

### 4.10 Histology and immunohistochemical analyses

After the treatment, the mice were sacrificed. The major tissues and tumors were separated for H&E staining, immunohistochemical analysis, and terminal deoxynucleotidyl transferase‐mediated dUTP nick‐end labeling (TUNEL) assays. The major organs and tumor tissues were fixed in 4% paraformaldehyde and embedded in paraffin. Then the specimens were sectioned at a thickness of 5 μm and stained with hematoxylin solution and eosin Y solution (H&E), Ki67, and TUNEL according to the manufacturer's instructions.

## CONFLICT OF INTEREST

The authors declare no competing financial interest.

## ETHICS STATEMENT

All animal experiments were approved by the Animal Ethics Committee of Shanghai Jiao Tong University (A2021030) and performed following the guidelines of care and use of laboratory animals.

## Supporting information



Supporting Information
