# Supplementary material for: Drug‐grafted DNA as a novel chemogene for targeted combinatorial cancer therapy
Source: Exploration (Beijing). 2022 Mar 22;2(2):20210172. doi: 10.1002/EXP.20210172 (PMC10190944; doi:10.1002/EXP.20210172)
Supplement: Supplementary file 1 — Supporting Information [file EXP2-2-20210172-s001.pdf]

Supporting Information

Drug-Grafted DNA as A Novel Chemogene for Targeted Combinatorial Cancer Therapy

Yuhe Liu[†1], Jiao Zhang[†1], Yuanyuan Guo[2], Ping Wang[3], Yue Su[1], Xin Jin[1], Xinyuan Zhu[1], Chuan Zhang*[1]

[1] School of Chemistry and Chemical Engineering, Frontiers Science Center for Transformative Molecules, Institute of Molecular Medicine, Sixth people's Hospital, School of Medicine, Shanghai Key Laboratory for Molecular Engineering of Chiral Drugs, Shanghai Jiao Tong University, Shanghai, 200240, China.

[2] Department of Radiology, Shanghai Jiao Tong University Affiliated Sixth People’s Hospital, Shanghai Jiao Tong University School of Medicine, 600 Yi Shan Road, Shanghai 200233, China.

[3] Weigao Research Center, 304, Building 2, Lane 720, Cailun Rd., Shanghai, China.

Correspondence

E-mail: chuanzhang@sjtu.edu.cn

[†] These authors contribute to this work equally

Experimental Section

1.1. Synthesis of disulfide-modified camptothecin (CPT-ss-OH)

CPT-ss-OH was synthesized according to the previously reported methods. [1, 2] Briefly, camptothecin (CPT) (1.000 g, 2.87 mmol) and triphosgene (0.315 g, 1.06 mmol) were suspended in anhydrous dichloromethane (DCM) (200 mL) and stirred under argon atmosphere. 4-(dimethylamino) pyridine (DMAP) (1.050 g, 8.60 mmol) dissolved in anhydrous DCM (10 mL) was then added dropwise into the mixture suspension, which was further stirred for 30 min. 2,2'-dithiodiethanol (4.420 g, 28.7 mmol) in anhydrous tetrahydrofuran (THF) (15 mL) was added into the mixture. After stirring overnight, the mixture was washed thrice with 0.1 M HCl solution (100 mL), and then washed twice with saturated brine (100 mL) and water (100 mL), respectively. The organic phase was dried over anhydrous Na_2SO_4 and the filtrate was concentrated under vacuum. The crude product was further purified by silica gel column chromatography using dichloromethane (CH_2Cl_2) and methanol (CH_2Cl_2/CH_3OH, 100:1 v: v) as the eluent. Yield: 1.41 g (64.5%). Liquid chromatography-mass spectrometry (LC-MS) m/z ($M+H^+$) calculated 529.106, found 529.103 ($M+H^+$). Proton nuclear magnetic resonance (^1H NMR, 400 MHz, d_6-DMSO) δ 8.67 (s, 1H), 8.15 (d, J = 8.6 Hz, 1H), 8.11 (d, J = 7.9 Hz, 1H), 7.85 (ddd, J = 8.4, 6.9, 1.3 Hz, 1H), 7.70 (dd, J = 11.1, 3.9 Hz, 1H), 7.03 (s, 1H), 5.56-5.38 (m, 2H), 5.34-5.19 (m, 2H), 4.38-4.19 (m, 2H), 3.62-3.46 (m, 2H), 3.02-2.88 (m, 2H), 2.80-2.64 (m, 2H), 2.30-2.01 (m, 2H), 0.98-0.84 (m, 3H).

1.2. Synthesis of carbonethyl bromide-modified CPT (CPT-ss-Br)

CPT-ss-OH (330 mg, 0.625 mmol) and N, N-diisopropylethylamine (DIEA) (88 mg, 0.687 mmol) were dissolved using anhydrous DCM (200 mL) under nitrogen. [2] Then, the solution of bromoacetyl bromide (126 mg, 0.625 mmol) was added under stirring and reacted overnight at room temperature. The solvent was evaporated by rotatory evaporator and the crude product was purified by silica gel column chromatography using CH_2Cl_2/CH_3OH (200:1, v: v) as the eluent. Yield: 327 mg (80.7%). LC-MS m/z ($M+H^+$) calculated 649.530, found 649.031 ($M+H^+$) and 651.0378($M+3H^+$). 1H NMR (400 MHz, $CDCl_3$) δ 8.41 (s, 1H), 8.21 (d, J = 8.5 Hz, 1H), 7.91 (d, J = 8.1 Hz, 1H), 7.90-7.71 (m, 1H), 7.66 (ddd, J = 8.0, 7.0, 1.0 Hz, 1H), 7.33 (s, 1H), 5.48 (dt, J = 27.5, 13.8 Hz, 2H), 5.31-5.22 (m, 2H), 4.48-4.20 (m, 4H), 3.87-3.72 (m, 2H), 3.01-2.80 (m, 4H), 2.38-2.04 (m, 2H), 0.99 (t, J = 7.5 Hz, 3H).

Supplementary Tables

Table S1. Oligonucleotide sequences used in this work.

Phosphorothioate oligonucleotides	Sequences
ASO-2PS	5'-TTT T TCT CCC* AGC GTG CGC* CAT-3'
Complementary strand of ASO	5'-ATG GCG CAC GCT T TTA GAA AAA-3'
Apt-ASO-2PS	5'- GGT GGT GGT GGT TGT GGT GGT GGT GG T TTT TCT CCC* AGC GTG CGC* CAT-3'
Apt-SC-2PS	5'- GGT GGT GGT GGT TGT GGT GGT GGT GG T TTT TCC CAC* CTC ACC TAC* CAT-3'
Cy5-ASO-2PS	5'-/Cy5/TTT T TCT CCC* AGC GTG CGC* CAT-3'
Cy5-Apt-ASO-2PS	5'- /Cy5/GGT GGT GGT GGT TGT GGT GGT GGT G G T TTT TCT CCC* AGC GTG CGC* CAT-3'
Cy5-Apt-SC-2PS	5'- /Cy5/GGT GGT GGT GGT TGT GGT GGT GGT G G T TTT TCC CAC* CTC ACC TAC* CAT-3'
Cy5.5-ASO-2PS	5'- /Cy5.5/TTT T TCT CCC* AGC GTG CGC* CAT-3'
Cy5.5-Apt-ASO-2PS	5'- /Cy5.5/GGT GGT GGT GGT TGT GGT GGT GGT GG T TTT TCT CCC* AGC GTG CGC* CAT-3'
Cy5.5-Apt-SC-2PS	5'- /Cy5.5/GGT GGT GGT GGT TGT GGT GGT GGT GG T TTT TCC CAC* CTC ACC TAC* CAT-3'

* represents the site with phosphorothioate modifications.

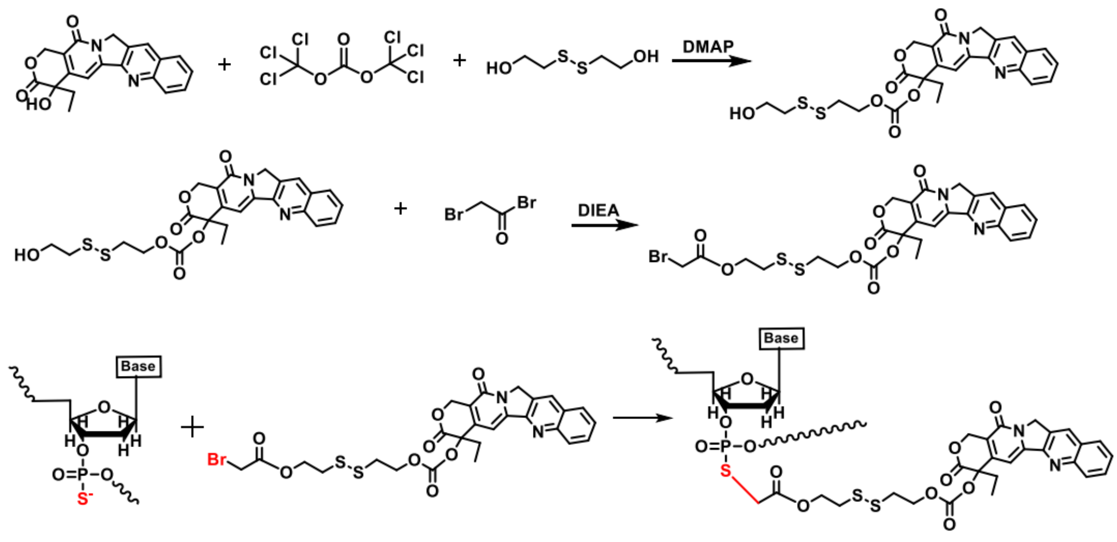

Scheme S1. The synthetic route of CPT-grafted oligonucleotide with a disulfide linkage.

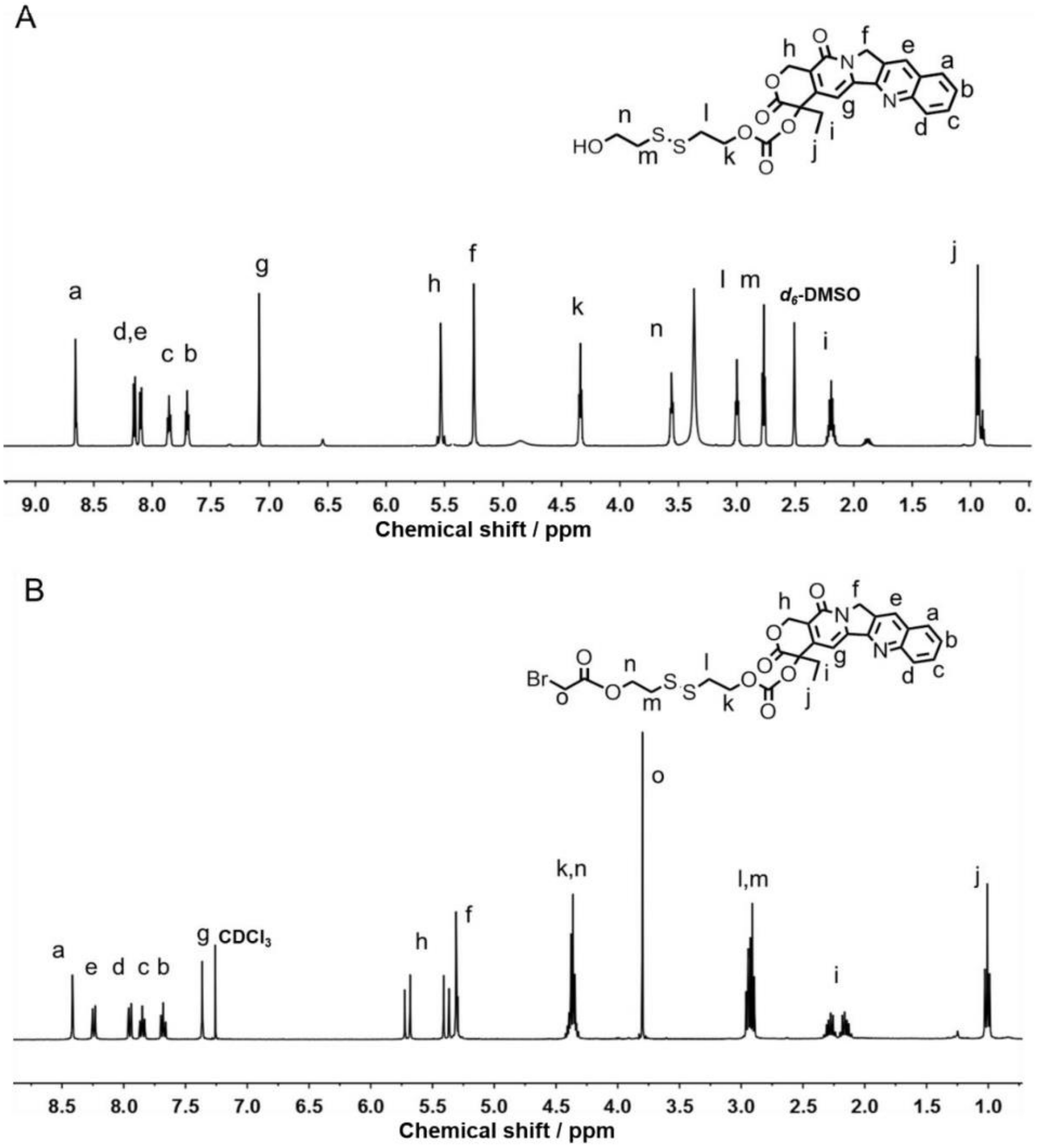

Figure S1. ^{1}H NMR spectra of CPT-ss-OH (A) and CPT-ss-Br (B).

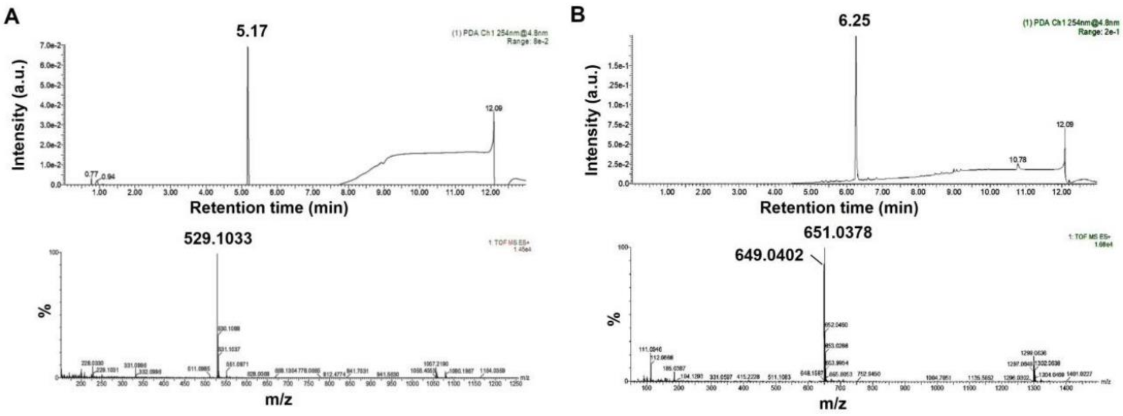

Figure S2. LC-MS spectra confirmed the successful syntheses of CPT-ss-OH (A) and CPT-ss-Br (B) with high purity.

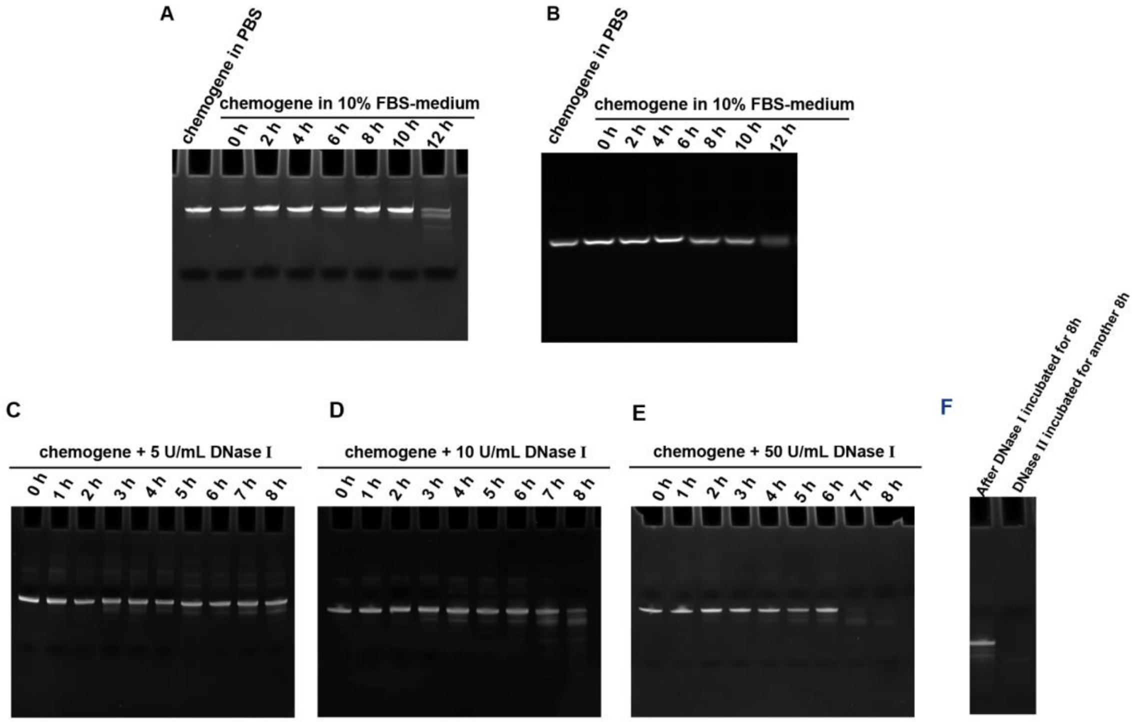

Figure S3. The stability of chemogene in 10% FBS-containing medium for different time as characterized by 20% denaturing polyacrylamide gel electrophoresis (A) and1% agarose gel electrophoresis (B). The stability of chemogene in the presence of 5 U/mL DNase I buffer (C), 10 U/mL DNase I (D), and 50 U/mL DNase I (E) respectively. After incubating for different time, samples were characterized by 10% denaturing

polyacrylamide gel electrophoresis. (F) The stability of chemogene in the presence of DNase II (20 U/mL). After 8 h incubation, samples were characterized by 10% denaturing polyacrylamide gel electrophoresis.

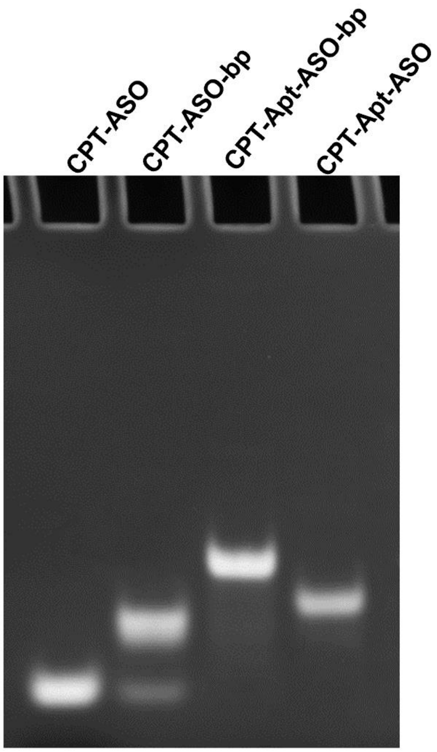

Figure S4. The molecular recognition between CPT-grafted ASOs and their complementary strands analyzed by 4% non-denaturing polyacrylamide gel electrophoresis.

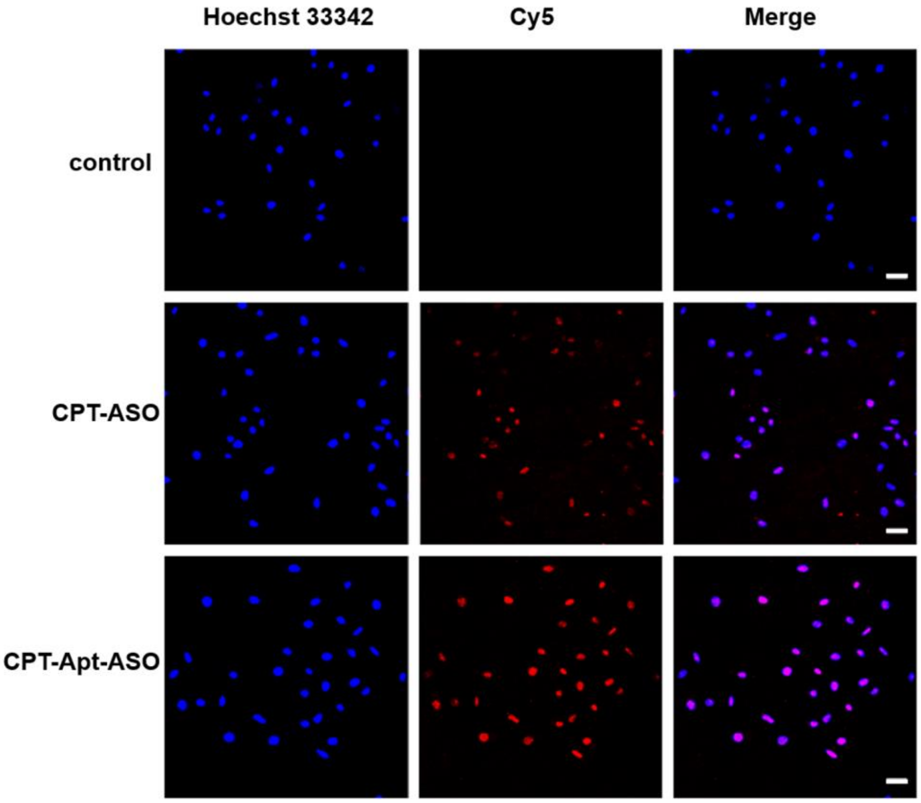

Figure S5. CLSM images of HeLa cells treated with Cy5-labeled CPT-ASO and Cy5-labeled CPT-Apt-ASO for 2 h. Blue: Hoechst (nuclear stain); red: Cy5. Scale bars: 50 μm.

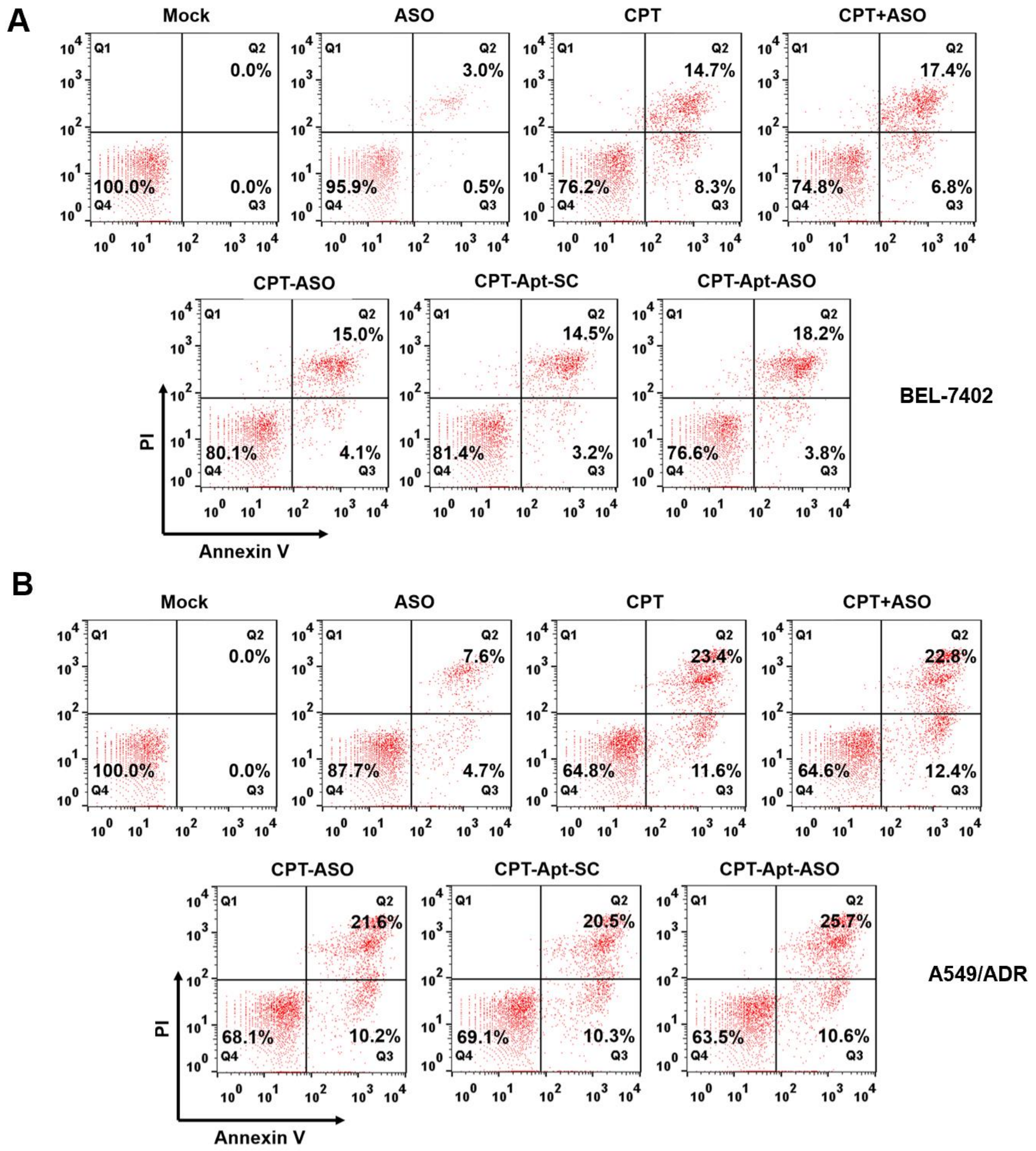

Figure S6. Flow cytometry-based apoptosis analysis of A) drug-resistant BEL-7402 cells and B) drug-resistant A549 cells induced by CPT, ASO, CPT+ASO, CPT-ASO, CPT-Apt-SC, and CPT-Apt-ASO respectively, at an equivalent CPT concentration of 0.2 μM for 48 h incubation.

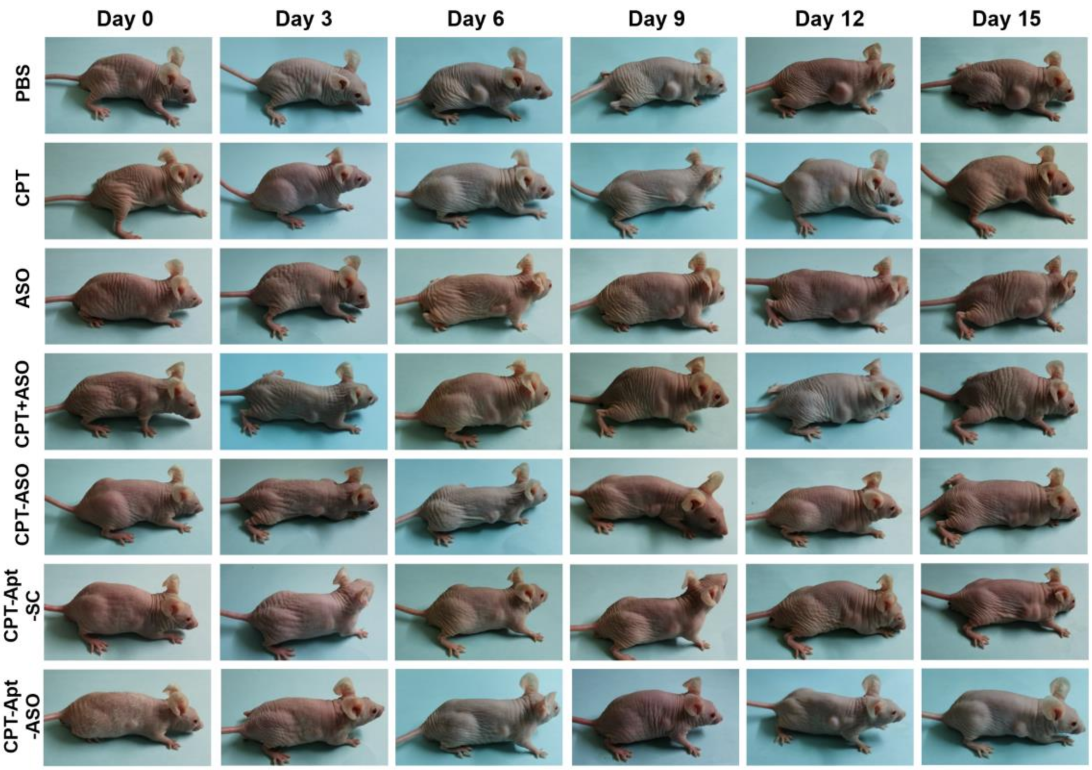

Figure S7. Photographs of the HeLa tumor-bearing nude mice treated with CPT, ASO, CPT+ASO, CPT-ASO, CPT-Apt-SC, and CPT-Apt-ASO during the experiment.

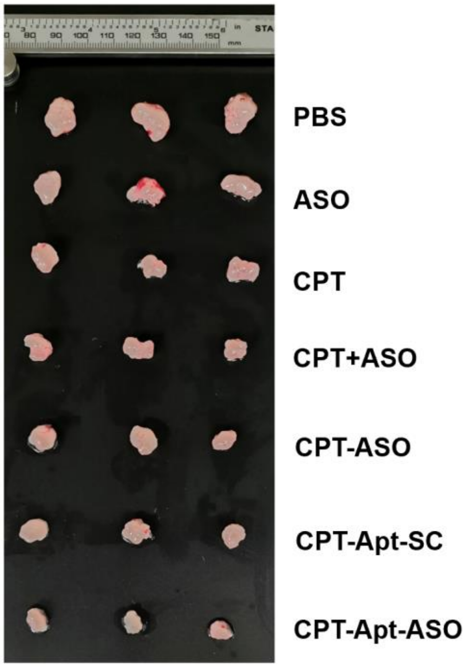

Figure S8. Images of tumors harvested from mice after three days of the last drug administration.

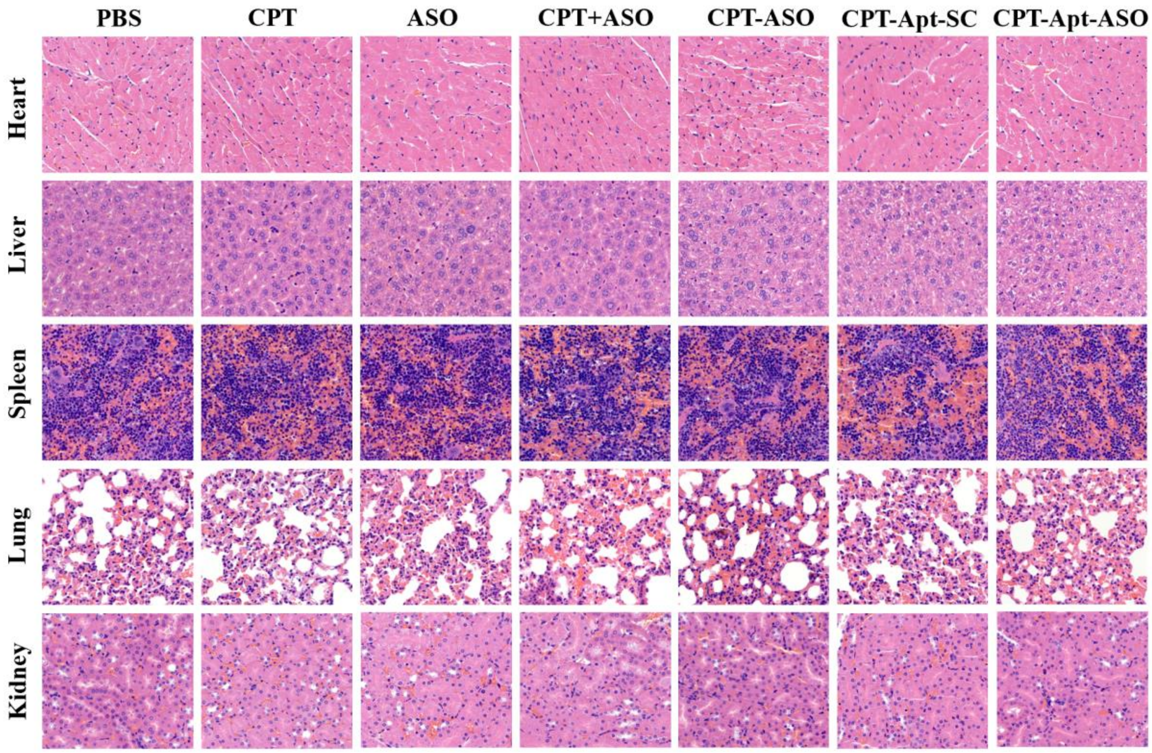

Figure S9. Histological examination of major organs of the HeLa tumor-bearing nude mice after anticancer treatments using different drug formulations.

References

[1] J. Liu, W. Liu, I. Weitzhandler, J. Bhattacharyya, X. Li, J. Wang, Y. Qi, S. Bhattacharjee, A. Chilkoti, *Angew. Chem. Int. Ed.*, **2015**, *54*, 1002.

[2] J. Zhang, Y. Guo, F. Ding, G. Pan, X. Zhu, C. Zhang, *Angew. Chem. Int. Ed.*, **2019**, *58*, 13794.
